# Arterial hypertension and associated factors: National Health Survey, 2019

**DOI:** 10.11606/s1518-8787.2022056004177

**Published:** 2022-11-18

**Authors:** Deborah Carvalho Malta, Regina Tomie Ivata Bernal, Edmar Geraldo Ribeiro, Alexandra Dias Moreira, Mariana Santos Felisbino-Mendes, Jorge Gustavo Velásquez-Meléndez

**Affiliations:** I Universidade Federal de Minas Gerais Escola de Enfermagem Departamento de Enfermagem Materno Infantil e Saúde Pública Belo Horizonte MG Brasil Universidade Federal de Minas Gerais. Escola de Enfermagem. Departamento de Enfermagem Materno Infantil e Saúde Pública. Belo Horizonte, MG, Brasil

**Keywords:** Hypertension, epidemiology, Risk Factors, Socioeconomic Factors, Health Status Disparities, Health Surveys

## Abstract

**OBJECTIVE:**

To analyze the factors associated with self-reported arterial hypertension, as well as its prevalence in the Brazilian adult population.

**METHODS:**

Data from 88,531 individuals aged 18 years or older who responded to the 2019 National Health Survey were analyzed. The outcome studied was self-reported arterial hypertension. Sociodemographic variables and clinical and lifestyle conditions were considered as exposures. The prevalence ratio (PR), crude and adjusted for sex, age, and schooling was used as a measure of association to verify the factors related to its prevalence, obtained by Poisson regression with robust variance.

**RESULTS:**

The prevalence of self-reported arterial hypertension was of 23.9% (95%CI: 23.4–24.4). When adjusting for age, sex, and schooling, the adjusted Prevalence Ratios (APR) were higher among: regular health self-assessment (APR = 1.6; 95%CI: 1.5–1.6) and bad health self-assessment (APR = 1.7; 95%CI: 1.6–1.8); self-reference to heart disease (APR = 1.7; 95%CI: 1.6–1.7), diabetes (APR = 1.7; 95%CI: 1.6–1.8), high cholesterol (APR = 1.6; 95%CI: 1.6–1.7), overweight (APR = 1.4; 95%CI: 1.4–1.5), and obesity (APR = 2.0; 95%CI: 1.9–2.1); high salt intake (APR = 1.1; 95%CI: 1.0–1.1); higher among former smokers (APR = 1.1; 95%CI: 1.1–1.2) and lower among smokers (APR = 0.9; 95%CI: 0.8–0.9); and consumption of ultra-processed foods (APR = 0.9; 95%CI: 0.8–0.9).

**CONCLUSION:**

A quarter of the Brazilian adult population claims to have arterial hypertension, more prevalent among women and associated with older age groups, Black, mixed-race, and others, low schooling, high salt intake, former smoking, presence of comorbidities, and worse health self-assessment.

## INTRODUCTION

Arterial hypertension is one of the leading causes of premature death worldwide. The World Health Organization (WHO) estimates that in 40 years the number of hypertensive patients has increased from 594 million people in 1975 to about 1.13 billion in 2015, two-thirds of whom live in low and middle income countries^1^.

The Global Burden of Diseases (GBD) estimates approximately 10.8 million deaths per year and 235 million disability-adjusted life years lost due to hypertension (DALYs – Disability-Adjusted Life Years)^2^.

It is a multifactorial clinical condition diagnosed when an increase in blood pressure levels is ≥ 140 and/or 90 mmHg, resulting from genetic/epigenetic, environmental, social, cultural, and lifestyle-related factors^3^. Among the modifiable risk factors of arterial hypertension, unhealthy diets stand out, characterized by excessive salt intake, a diet rich in saturated and trans fats, low consumption of fruits and vegetables; sedentary lifestyle; consumption of tobacco and alcohol; and overweight or obesity^3^. Non-modifiable risk factors include a family history of hypertension, age over 65 years, and coexisting diseases such as diabetes mellitus or kidney disease^1,3^.

Hypertension results in a significant impact on medical and socioeconomic costs due to complications in the target organs, such as the heart (coronary artery disease), heart failure, atrial fibrillation and sudden death; brain (ischemic or hemorrhagic stroke), dementia; kidneys (chronic kidney disease); and arterial system (peripheral obstructive arterial disease)^3^.

Prevention and early detection measures are effective to control hypertension and include healthy lifestyles such as: cessation of tobacco use, reduction of salt intake, consumption of fruits and vegetables, regular physical activity, prevention of harmful alcohol use, and weight control. Regarding primary health care, pharmacological interventions should be evaluated^1,3^.

Reducing the prevalence of arterial hypertension is one of the WHO’s global goals in the Action Plan for the Prevention and Control of Noncommunicable Diseases^4^, which is a major challenge, given the aging population and difficulties in controlling risk factors. Therefore, it is highly relevant to conduct population-based surveys that allow for this disease’s continuous monitoring and surveillance in the Brazilian population, its distribution, affected populations, and factors related to this condition, which may support public policies for prevention and health promotion^4^. The last studies that allowed such analysis on a national scope were based on population measures carried out almost a decade ago, in 2013^5^. We should note that, although the Vigitel (Surveillance System for Risk and Protective Factors for Chronic Diseases) by Telephone Survey estimates annually the prevalence of arterial hypertension, these data refer only to the population living in the capitals of the federative units^6^.

Thus, this study aims to analyze the factors associated with self-reported arterial hypertension, as well as its prevalence, in the Brazilian adult population.

## METHODS

### Study Design

This study used data from the National Health Survey (PNS), conducted in 2019 by the Brazilian Institute of Geography and Statistics (IBGE), in partnership with the Ministry of Health. Research is scheduled to take place every five years and allows the monitoring of risk and protective factors for chronic non-communicable diseases, including high blood pressure, lifestyles, and sociodemographic factors^7^.

The samples were taken in three stages, and in the first stage the primary sampling units were drawn, consisting of census tracts, or set of sectors. In the second stage, private households were randomly drawn in the selected units and, in the third stage, a resident with 15 years or more was randomly drawn. Details about the sampling process, design, and realization of the PNS 2019 are published^7^.

The expected sample of the PNS 2019 was 108,525 households and data were collected in 94,114 households. For this study’s analyses, only the interviews of residents aged 18 years and over were considered, totaling 88,531 individuals, the population of interest in this study.

### Variables

The outcome studied was arterial hypertension, obtained by questions from the non-communicable chronic diseases module of the PNS: “Has any doctor ever diagnosed you with arterial hypertension (high blood pressure)?” – Hypertension Q2a +Q2b.

As exposures, the following variables were analyzed according to the IBGE/PNS Report, 2019^7^:

a.*Sociodemographic characteristics:*

- Sex: male and female;
- Age group: 18–24, 25–39, 40–59, and ≥ 60;
- Education: illiterate and incomplete elementary school, complete elementary school and incomplete middle school, complete middle school0 and incomplete higher education, and complete higher education;
- Race/color: white, mixed-race, Black, and others (Asian and Indigenous);
- Income in minimum wages (mw): up to 1 mw, 1 to 3 mw, 3 or more mw;

b.*Health conditions:*

- Self-assessment of health status: good/very good, regular, and bad/very bad;
- Self-reported diagnosis of heart disease: yes, no;
- Self-reported diagnosis of diabetes: yes, no;
- Self-reported diagnosis of high cholesterol: yes, no;
- Nutritional status: eutrophic (BMI < 25 kg/m^2^), overweight (BMI between 25 and 29 kg/m^2^), and obese (BMI ≥ 30 kg/m^2^). Body mass index (BMI) was calculated from the report of weight and height measurements.

c.*Lifestyle:*

- Smoking: non-smoker, former smoker, and smoker;
- Abusive alcohol consumption: yes, no. Abusive consumption was considered when consuming five or more doses in a single occasion.
- Consume five or more groups of minimally or non-processed foods protective against chronic diseases the day before the interview such as: (rice; cassava; beans, peas; beef, pork, chicken, or fish; egg; lettuce, cabbage, broccoli; pumpkin, carrots; tomatoes and vegetables; papaya, mango; orange and fruits; milk; peanuts, chestnuts);
- Consumption of ultra-processed foods: yes or no. We considered the consumption report of five or more groups of industrialized foods the day before the interview: (soda; juice in a box; chocolate drink; cream crackers; sweet or stuffed cookies; industrialized dessert; bologna or ham; white bread; industrialized sauces; instant noodles);
- High salt intake: yes, no. It was considered high when individuals answered “Too high” or “High” to the question: “Considering freshly prepared food and processed foods, you think your salt consumption is...”
- Sufficient leisure time physical activity (LTPA): yes; no. Individuals who reported practicing at least 150 minutes of mild or moderate intensity or 75 minutes of vigorous intensity were considered as active during leisure time.

### Statistical Analysis

First, the prevalence of arterial hypertension and 95% confidence intervals (95%CI) were estimated, according to sociodemographic and health characteristics. To verify the factors associated with this prevalence, the prevalence ratio (PR), crude and adjusted by sex, age, race/color, and schooling, was used as a measure of association, obtained by Poisson regression with robust variance.

All analyses were performed in the *Data Analysis and Statistical Software (Stata)* software version 14, using the *survey* module that considers the post-stratification weights.

### Ethical Aspects

The project of the PNS was forwarded to the National Research Ethics Commission of the National Health Council and approved under Opinion No. 3,529,376, issued on August 23, 2019. This study used publicly available secondary data of the PNS, without identifying the subjects, exempted from consideration by the research ethics committee, in accordance with CNS Resolution No. 466/2012.

## RESULTS

The prevalence of self-reported arterial hypertension, according to previous medical diagnosis, was of 23.9% (95%CI: 23.5–24.4), and was higher among women (26.4%; 95%CI: 25.8–27.2), people aged 60 years or older (55.0%; 95%CI: 53.9–56.1), the population with low schooling (36.6%; 95%CI: 35.7–37.5) and Black race/color (25.8%; 95%CI: 24.4–27.2), and among people with diverse characteristics 27.7% (95%CI: 23.3–32.2). Prevalence rates were higher in the group with income between one and three minimum wages (Table 1).

**Table 1:** Prevalence and confidence interval (95%) for hypertension according to sociodemographic characteristics. National Health Survey, Brazil, 2019.

Sociodemographic characteristics	Self-reported hypertension								

	%	(95%CI)		PR	(95%CI)		APR	(95%CI)	
Total	23.93	23.42	23.93						
Male	21.06	20.37	21.06	1.00			1.00		
Female	26.45	25.75	26.45	1.26	1.21	1.31	**1.18**	**1.14**	**1.22**
Age group (years)									
18–24	2.29	1.73	2.85	1.00			1.00		
25–39	7.25	6.69	7.81	3.16	2.44	4.10	**3.21**	**2.47**	**4.16**
40–59	27.21	26.28	28.14	11.88	9.27	15.22	**11.52**	**8.98**	**14.77**
≥ 60	54.99	53.92	56.06	24.00	18.77	30.70	**22.17**	**17.31**	**28.39**
Schooling									
Illiterate and incomplete primary education	36.55	35.65	37.45	1.00			1.00		
Complete primary education and incomplete secondary education	20.38	19.13	21.63	0.56	0.52	0.60	0.96	0.91	1.02
Complete secondary education and incomplete higher education	15.44	14.71	16.17	0.42	0.40	0.45	**0.84**	**0.80**	**0.88**
Complete higher education	18.17	17.07	19.27	0.50	0.47	0.53	**0.74**	**0.70**	**0.79**
Race/color									
White	24.36	23.56	25.16	1.00			1.00		
Mixed-race	22.88	22.18	23.58	0.94	0.90	0.98	**1.05**	**1.01**	**1.09**
Black	25.81	24.42	27.19	1.06	1.00	1.13	**1.15**	**1.08**	**1.22**
Others (Asian/Indigenous)	27.74	23.25	32.22	1.14	0.97	1.34	**1.15**	**1.02**	**1.30**
Health plan									
No	23.77	23.20	24.34	1.00			1.00		
Yes	24.34	23.35	25.34	1.02	0.98	1.07	**1.07**	**1.02**	**1.12**
Income (mw)									
< 1	22.42	21.76	23.07	1.00			1.00		
≥ 1–3	25.64	24.80	26.48	1.14	1.10	1.19	1.00	0.97	1.04
> 3	25.15	23.63	26.66	1.12	1.05	1.20	1.02	0.95	1.09

PR: prevalence ratio; APR: prevalence ratio adjusted for gender, age, and schooling; MW: minimum wage.

Values with statistical significance are shown in bold.

Prevalence ratios (PR) adjusted for sex, education, and age were: female (APR = 1.2; 95%CI: 1.1–1.2); age group – reference: 18 to 24, 25 to 39 years (APR = 3.2; 95%CI: 2.5–4.2), 40 to 59 years (APR = 11.5; 95%CI: 9.0–14.8), 60 and older (APR = 22.2; 95%CI: 17.3–28.4); education – reference: illiterate and incomplete elementary school, with lower prevalence of hypertension in the higher levels of schooling, for the population with complete high school and incomplete higher education (APR = 0.8; 95%CI: 0.8–0.9), complete higher education (APR = 0.7; 95%CI: 0.7–0.8); assuming the white race/color as a reference, the highest prevalence of hypertension was among Black people (APR = 1.1; 95%CI: 1.1–1.2), mixed-race people (APR = 1.0; 95%CI: 1.0–1.1), others (APR = 1.2; 95%CI: 1.0–1.3); those with health insurance (APR = 1.1; 95%CI: 1.0–1.1). We found no difference in prevalence and income (Table 1).

Table 2 shows the prevalence of hypertension, according to clinical conditions. The prevalence of hypertension was higher among: those who define their health situation as regular and bad at 38.2% (95%CI: 37.2–39.3), and 49.8% (95%CI: 47.6–52.0), respectively; those diagnosed with heart disease 61.2% (95%CI: 59.0–64.0), diabetes 63.8% (95%CI: 61.9–65.7), high cholesterol 50.9% (95%CI: 49.4–52.3), overweight 25.7% (95%CI: 24.9–26.5), and obesity 37.2% (95%CI: 36.0–38.4).

**Table 2:** Prevalence and confidence interval (95%) for hypertension according to clinical conditions. National Health Survey, Brazil, 2019.

Lifestyle	Self-reported hypertension								

	%	(95%CI)		PR	(95%CI)		APR	(95%CI)	
Smoking									
Non-smoker	20.59	19.98	21.20	1.00			1.00		
Former smoker	32.91	31.85	33.96	1.60	1.53	1.67	**1.12**	**1.08**	**1.16**
Smoker	21.04	19.66	22.43	1.02	0.95	1.09	**0.88**	**0.82**	**0.94**
Five or more protective food groups									
No	23.30	22.73	23.88	1.00			1.00		
Yes	25.93	24.92	26.93	1.11	1.06	1.16	0.98	0.94	1.02
Five or more ultra-processed food groups									
No	25.53	24.97	26.10	1.00			1.00		
Yes	14.30	13.23	15.37	0.56	0.52	0.61	**0.86**	**0.80**	**0.93**
Abusive consumption of alcohol									
No	25.56	24.99	26.13	1.00			1.00		
Yes	15.98	14.89	17.06	0.63	0.58	0.67	1.03	0.97	1.10
High salt intake									
No	24.61	24.07	25.15	1.00			1.00		
Yes	19.23	17.91	20.55	0.78	0.73	0.84	**1.08**	**1.01**	**1.14**
Leisure time physical activity									
No	26.22	25.61	26.83	1.00			1.00		
Yes	18.44	17.56	19.32	0.70	0.67	0.74	0.98	0.93	1.03

PR: prevalence ratio; APR: prevalence ratio adjusted for gender, age, and schooling.

Values with statistical significance are shown in bold.

When adjusting for age, sex, and schooling, the APR were higher among: those who define their health situation as regular (APR = 1.6; 95%CI: 1.5–1.6) and bad (APR = 1.7; 95%CI: 1.6–1.8); those who claim to have heart disease (APR = 1.7; 95%CI: 1.6–1.7), diabetes (APR = 1.7; 95%CI: 1.6–1.8), high cholesterol (APR = 1.6; 95%CI: 1.6–1.7); overweight (APR = 1.4; 95%CI: 1.4–1.5); and obesity (APR = 2.0; 95%CI: 1.9–2.1).

Table 3 shows the prevalence of arterial hypertension according to lifestyles: among former smokers 32.9% (95%CI: 31.9–34.0), smokers 21.0% (95%CI: 19.7–22.4); those who consume healthy foods 25.9% (95%CI: 24.9–26.9), and was lower among those who consume ultra-processed foods: 14.3% (95%CI: 13.2–15.4); among those who abuse alcohol consumption 16.0% (95%CI: 15.0–17.0); those with high salt intake 19.2% (95%CI: 18.0–20.6), and those who practice physical activity 18.4% (95%CI: 17.6–19.3).

**Table 3:** Prevalence and confidence interval (95%) for hypertension according to clinical conditions. National Health Survey, Brazil, 2019.

Clinical conditions	Self-reported hypertension								

	%	(95%CI)		PR	(95%CI)		APR	(95%CI)	
Health assessment									
Good/very good	16.06	15.54	16.59	1.00			1.00		
Regular	38.23	37.22	39.25	2.38	2.28	2.48	**1.56**	**1.50**	**1.62**
Bad/very bad	49.77	47.57	51.97	3.10	2.94	3.27	**1.74**	**1.65**	**1.83**
Heart disease									
No	21.84	21.33	22.35	1.00			1.00		
Yes	61.23	58.96	63.50	2.80	2.68	2.93	**1.67**	**1.60**	**1.74**
Diabetes									
No	20.59	20.10	21.08	1.00			1.00		
Yes	63.75	61.85	65.66	3.10	2.98	3.21	**1.69**	**1.63**	**1.76**
High cholesterol^a^									
No	20.50	19.97	21.03	1.00			1.00		
Yes	50.85	49.39	52.32	2.48	2.39	2.57	**1.63**	**1.57**	**1.69**
Nutritional status^b^									
Eutrophic	16.07	15.40	16.74	1.00			1.00		
Overweight	25.69	24.86	26.51	1.60	1.52	1.69	**1.42**	**1.36**	**1.49**
Obesity	37.20	36.01	38.39	2.32	2.20	2.44	**2.02**	**1.92**	**2.12**

PR: prevalence ratio; APR: prevalence ratio adjusted for gender, age, and schooling.

^a^ 7.280 missing.

^b^ 853 missing.

Values with statistical significance are shown in bold.

When adjusting for age, sex, and schooling, the APR were higher among: those with high salt intake (APR = 1.1; 95%CI: 1.0–1.1); ex-smokers (APR = 1.1; 95%CI: 1.1–1.2); and lower among smokers (APR = 0.9; 95%CI: 0.8–0.9); those who consume ultra-processed foods (APR = 0.9; 95%CI: 0.8–0.9). The associations between consumption of healthy foods, alcohol abuse, and sufficient practice of physical activity with hypertension were not significant in the adjusted model.

## DISCUSSION

About a quarter of the population self-reported arterial hypertension, being higher among women, older age groups, in the population with low schooling, among Black and mixed-race people, and is also associated with comorbidities such as: heart disease, diabetes, high cholesterol, overweight, obesity, and people who define their health as regular or poor. Among lifestyles, the highest prevalence was among former smokers, those who reported high salt intake, and was lower among smokers and those who abuse alcohol consumption.

The self-reported prevalence of the PNS 2013 was lower, 21.4% (95%CI: 20.8–22.0)^9^, than what we found in our study, which can be explained by population aging, as well as the increased prevalence of obesity and overweight in the population in the period.

Regarding sociodemographic factors, we observed a higher prevalence of hypertension among women. According to a study^9^, women tend to seek health services more than men, which gives them greater opportunities for a diagnosis, and that may have been reflected in the prevalence of higher hypertension among female respondents. However, when using blood pressure measurements, the PNS 2013 identified the opposite: higher pressure among men^10^, which was also observed in a meta-analysis conducted from 1,479 studies, representing the blood pressure measurement of 19.1 million adults, a number estimated by the global study group in 2015 (NCD Risk Factor Collaboration), which estimated a prevalence of hypertension in 24.1% of men (95%CI: 21.4–27.1) and in 20.1% of women (95%CI: 17.8–22.5)^8^.

The literature emphasizes that arterial hypertension increases with age, as it is associated with aging and its progressive hardening of blood vessels^1,3,11^. It is noteworthy that age was the factor of greatest magnitude of association in this study.

The study identified, after adjustments by age, sex, and schooling, a higher prevalence among Black, mixed-race, Asians, and Indigenous people. The literature emphasizes that arterial hypertension may result from a genetic predisposition in Black people, besides being a population more socially exposed to the risk factors inherent to hypertension^12^.

Regarding socioeconomic characteristics, populations with low schooling had a higher incidence of hypertension. Among the reasons for this result, many studies point to exposure to vulnerabilities and stressors, worse socioeconomic conditions, lack of access to health services, and less access to guidance, health promotion actions, healthy eating, and health care^13^. However, after controlling the adjustment variables, the income showed no association with the clinical condition focused on this study. People who hired health plans presented the opposite result, which had already been indicated in other studies in Brazil^14^. Having a paid health plan facilitates access to health services, which provides greater opportunity for diagnosis.

Factors that affect the population’s lifestyle, such as tobacco use, are considered causing risks for cardiovascular diseases^15^and, to avoid such diseases, avoiding these substances is the first recommendation for their prevention^3^. The association between smoking cessation and hypertension has been described in the literature^11^ and can be both the effect of the study’s cross-sectional design and the abandonment of addiction after medical guidance from diagnosis^3^. However, alongside weight gain, smoking cessation is described as a possible stressful factor, which may contribute to increase the risk of hypertension.

Another important risk factor for the prevalence of hypertension is salt abuse, which this study confirmed, even after adjustments. The general recommendation for salt intake is 2g or 5g of sodium per day^16^. A meta-analysis study conducted in China showed that replacing table salt with potassium chloride can bring benefits in lowering systolic blood pressure (-5.7mmHg; 95%CI: -8.5 to -2.8) and diastolic blood pressure (-2.0mmHg; 95%CI: -3.5 to -0.4)^17^.

Longitudinal studies and systematic reviews^18^indicate that a diet rich in fruits, vegetables, grains, and low fat may result in a reduction in blood pressure and adhering to the diet with higher content of fruits and vegetables is associated with lower risk of stroke^18^, cardiovascular mortality^19^, and chronic kidney disease^20^. Likewise, all the evidence in the literature indicates that foods with high fat content, salt, and additives increase the prevalence of cardiovascular diseases and chronic non-communicable diseases^16^. However, this study found that people with an unhealthy diet would have a lower prevalence of hypertension, an unexpected result. However, the complementary analyses that were not shown showed that the consumption of ultra-processed foods was up to four times higher in younger populations, aged between 18 and 34 years, compared to the older populations, who in turn have a prevalence of hypertension up to 20 times higher. Therefore, the effect persisted even when adjusting for age and other variables of the model, possibly due to the strong association of this type of food among young people and the limits of cross-sectional studies, in causality analyses^21^.

Likewise, the literature makes it clear that regular physical activity (150 min/week) and reduction of sedentary behavior reduce arterial hypertension^22^. We estimate that excessive alcohol consumption is responsible for about 10% to 30% of hypertension cases and approximately 6% of all-cause mortality worldwide^23,24^. However, we did not find in this study the associations with physical activity, healthy eating, and alcohol, which have also been described in other cross-sectional studies, analyzing the results of telephone surveys and hypertension^7^. The fact that younger populations have a higher frequency of physical activity, alcohol, and ultra-processed foods may justify this negative result, even after adjusting for age.

Hypertension is the main modifiable risk factor associated with cardiovascular diseases, chronic heart disease, and premature death. It is associated with metabolic risk factors for diseases of the cardiocirculatory and renal systems, such as dyslipidemia, abdominal obesity, and DM^3^.

This study also pointed out that comorbidities, such as obesity, were the variable most strongly associated with arterial hypertension. The literature points to a direct, continuous, and almost linear relation between excess weight (overweight/obesity) and high blood pressure levels^25^. Obesity, especially visceral obesity, is an important risk factor for the elevation of hypertension and may be responsible for 65% to 75% of high blood pressure cases^26^. One of the most important measures in disease control is weight reduction, as identified by a meta-analysis including 25 studies, indicating that the loss of 5.1 kg reduced, on average, systolic blood pressure by 4.4 mmHg and diastolic blood pressure by 3.6 mmHg^25^.

Studies have shown that increased blood pressure results in increased risks of heart failure, atrial fibrillation, valvular heart diseases and peripheral arterial disease^24^, chronic kidney disease^24^, dementia and Alzheimer’s disease^27^, and probably diabetes mellitus^28^. These outcomes occur due to many years of exposure to high blood pressure levels^24^. The current study, although cross-sectional, confirmed the association with diabetes and cardiovascular disease, high cholesterol, and obesity.

Bad or very bad self-assessment of health was strongly associated with arterial hypertension. Studies point to this indicator as a predictor of worse health and mortality outcomes^29^.

Among this study’s limitations, because it is a cross-sectional study design, are the measurements of risk or protective factors and outcomes that are performed simultaneously, which limits inferences about the directionality of some associations in the causal model. We emphasize the bias of reverse causality or changes in lifestyles, motivated by the disease’s diagnosis and the guidance of health professionals. The use of self-reported morbidity data is dependent on access to health services for diagnosis; thus, those who use the service more frequently have a greater opportunity of a medical diagnosis of hypertension. On the other hand, because it is the most important health research in the country, the data allow this disease’s monitoring in the Brazilian population.

In short, the results estimate that about a quarter of the Brazilian adult population reported having hypertension, with the most prevalent outcome in women and older age groups, with Black and mixed people, low schooling, high salt intake, ex-smoking, presence of comorbidities, and worse self-assessment of health. Knowing these factors can help in the prevention, identification, management, and control of hypertension and chronic non-communicable diseases.
