# Supplementary material for: Arterial hypertension and associated factors: National Health Survey, 2019
Source: Rev Saude Publica. 2022 Nov 18;56:122. doi: 10.11606/s1518-8787.2022056004177 (PMC9749662; doi:10.11606/s1518-8787.2022056004177)
Supplement: Translation

# Translation: Hipertensão arterial e fatores associados: Pesquisa Nacional de Saúde, 2019

**Authors:** Deborah Carvalho Malta, Regina Tomie Ivata Bernal, Edmar Geraldo Ribeiro, Alexandra Dias Moreira, Mariana Santos Felisbino-Mendes, Jorge Gustavo Velásquez-Meléndez

**Affiliations:** I Brasil Universidade Federal de Minas Gerais. Escola de Enfermagem. Departamento de Enfermagem Materno Infantil e Saúde Pública. Belo Horizonte, MG, Brasil

**Keywords:** Hipertensão, epidemiologia, Fatores de Risco, Fatores Socioeconômicos, Disparidades nos Níveis de Saúde, Inquéritos Epidemiológicos

## Abstract

**OBJETIVO:**

Analisar os fatores associados à hipertensão arterial autorreferida, bem como sua prevalência, na população de adultos brasileiros.

**MÉTODOS:**

Foram analisados dados de 88.531 indivíduos de 18 anos ou mais que responderam à Pesquisa Nacional de Saúde de 2019. O desfecho estudado foi a hipertensão arterial autorreferida. Como exposições, foram consideradas variáveis sociodemográficas, condições clínicas e de estilo de vida. Para verificar os fatores associados à prevalência, usou-se como medida de associação a razão de prevalência (RP) bruta e ajustada por sexo, idade e escolaridade, obtidas por meio da Regressão de Poisson com variância robusta.

**RESULTADOS:**

A prevalência da hipertensão arterial autorreferida foi de 23,9% (IC95% 23,4–24,4). Ao ajustar por idade, sexo e escolaridade, as Razões de Prevalência ajustadas (RPaj) foram mais elevadas entre: auto avaliação de saúde regular (RPaj = 1,6; IC95% 1,5–1,6) e ruim (RPaj = 1,7; IC95% 1,6–1,8); autorreferência a doença do coração (RPaj = 1,7; IC95% 1,6–1,7), diabetes (RPaj = 1,7; IC95% 1,6–1,8), colesterol elevado (RPaj = 1,6; IC95% 1,6–1,7), sobrepeso (RPaj = 1,4; IC95% 1,4–1,5) e obesidade (RPaj = 2,0; IC95% 1,9–2,1); consumo elevado de sal (RPaj = 1,1; IC95% 1,0–1,1); entre ex-fumantes (RPaj = 1,1; IC95% 1,1–1,2) e menor entre fumantes (RPaj = 0,9; IC95% 0,8–0,9) e consumo de alimentos ultraprocessados (RPaj = 0,9; IC95% 0,8–0,9).

**CONCLUSÃO:**

Um quarto da população adulta brasileira afirma ter hipertensão arterial, de forma mais prevalente entre as mulheres e associada às maiores faixas etárias, cor da pele/raça preta, parda e outras, baixa escolaridade, consumo elevado de sal, ex-tabagismo, presença de comorbidades e pior autoavaliação de saúde.

## INTRODUÇÃO

A hipertensão arterial se constitui uma das principais causas de morte prematura em todo o mundo. A Organização Mundial de Saúde (OMS) estima que em 40 anos o número de hipertensos deu um salto, dos 594 milhões de pessoas, em 1975, para cerca de 1,13 bilhões, em 2015, sendo que dois terços deles vivem em países de baixa e média renda^1^.

Estudos do Global Burden of Diseases (GBD) estimam cerca de 10,8 milhões de mortes anuais e 235 milhões de anos de vidas perdidos ajustados por incapacidade devido à hipertensão arterial (DALYs – *Disability Adjusted Life Years*)^2^.

Trata-se de uma condição clínica multifatorial diagnosticada quando há elevação sustentada dos níveis pressóricos ≥ 140 e/ou 90 mmHg, resultante de fatores genéticos/epigenéticos, ambientais, sociais, culturais e relacionados aos estilos de vida^3^. Dentre os fatores de risco modificáveis da hipertensão arterial destacam-se as dietas não saudáveis, caracterizadas pelo consumo excessivo de sal, dieta rica em gordura saturada e gorduras trans, baixo consumo de frutas e vegetais; sedentarismo; consumo de tabaco e álcool e excesso de peso ou obesidade^3^. Os fatores de risco não modificáveis incluem histórico familiar de hipertensão, idade acima de 65 anos, além de doenças coexistentes, como diabetes mellitus ou doença renal^1,3^.

A hipertensão arterial resulta em impacto significativo nos custos médicos e socioeconômicos em decorrência das complicações nos órgãos-alvo, como o coração (doença arterial coronária), insuficiência cardíaca, fibrilação atrial e morte súbita; cérebro (acidente vascular encefálico isquêmico ou hemorrágico), demência; rins (doença renal crônica) e sistema arterial (doença arterial obstrutiva periférica)^3^.

Medidas de prevenção e de detecção precoce são ações efetivas para o controle da hipertensão e incluem estilos de vida saudáveis como: a cessação do uso do tabaco, redução do sal na dieta, consumo de frutas e vegetais, atividade física regular, prevenção do uso nocivo de álcool e atitudes para controle do peso. No âmbito da atenção primária à saúde deve-se avaliar a necessidade de intervenções farmacológicas^1,3^.

Reduzir a prevalência de hipertensão arterial constitui uma das metas globais da OMS no Plano de Enfrentamento das Doenças Crônicas Não Transmissíveis^4^, o que representa um grande desafio, dado o envelhecimento populacional e as dificuldades no controle dos fatores de risco. Portanto, torna-se de grande relevância a realização de pesquisas de base populacional que permitam o monitoramento contínuo e a vigilância desse agravo na população brasileira, sua distribuição, populações afetadas e fatores relacionados a essa condição, o que poderá subsidiar políticas públicas de prevenção e promoção à saúde^4^. Os últimos estudos que permitiram tal análise em abrangência nacional partiram de medidas populacionais realizadas há quase uma década, em 2013^5^. Vale ressaltar que, embora o Vigitel (Vigilância de Fatores de Risco e Proteção para Doenças Crônicas) por Inquérito Telefônico, estime anualmente a prevalência de hipertensão arterial, estes dados referem-se apenas à população residente nas capitais das Unidades Federativas^6^.

Assim, o estudo visa analisar os fatores associados à hipertensão arterial autorreferida, bem como sua prevalência, na população de adultos brasileiros.

## MÉTODOS

### Desenho do Estudo

Neste estudo foram utilizados os dados da Pesquisa Nacional de Saúde (PNS), realizada em 2019 pelo Instituto Brasileiro de Geografia e Estatística (IBGE), em parceria com o Ministério da Saúde. A pesquisa está programada para acontecer a cada cinco anos e possibilita o monitoramento dos fatores de risco e proteção para doenças crônicas não transmissíveis, dentre elas a pressão arterial elevada, estilos de vida e fatores sociodemograficos^7^.

As amostras foram tomadas em três estágios, sendo no primeiro sorteadas as unidades primárias de amostragem, constituídas por setores censitários ou conjunto de setores. No segundo estágio, foram aleatoriamente sorteados domicílios particulares nas unidades selecionadas e, no terceiro estágio, em cada domicílio foi sorteado um morador com 15 anos ou mais. Detalhes sobre o processo amostral, o desenho e realização da PNS 2019 encontram-se publicados^7^.

A amostra prevista da PNS 2019 foi de 108.525 domicílios e os dados foram coletados em 94.114 domicílios. Para as análises do presente estudo, considerou-se apenas as entrevistas de moradores com 18 anos e mais, totalizando 88.531 indivíduos, população de interesse deste estudo.

### Variáveis

O desfecho estudado foi hipertensão arterial, obtido por meio das perguntas do módulo de doenças crônicas não transmissíveis da PNS: “Algum médico já lhe deu o diagnóstico de hipertensão arterial (pressão alta)?” – Hipertensão Q2a +Q2b.

Como exposições, foram analisadas as seguintes variáveis, de acordo com o Relatório do IBGE/PNS, 2019^7^:

a)*Características sociodemográficas:*

- Sexo: masculino e feminino;
- Faixa etária: 18–24, 25–39, 40–59 e ≥ 60;
- Escolaridade: sem instrução e fundamental completo, fundamental completo e médio incompleto, médio completo e superior incompleto e superior completo;
- Raça/cor: branca, parda, preta e outras (amarela e indígena);
- Renda em salários-mínimos (sm): até 1 sm, 1 a 3 sm, 3 ou mais sm;

b)*Condições de saúde:*

- Autoavaliação do estado de saúde: bom/muito bom, regular e ruim/muito ruim;
- Diagnóstico autorreferido de doença cardíaca: sim, não;
- Diagnóstico autorreferido diabetes: sim, não;
- Diagnóstico autorreferido de colesterol alto: sim, não;
- Estado nutricional: eutróficos (IMC < 25 kg/m^2^), com sobrepeso (IMC entre 25 e 29 kg/m^2^) e com obesidade (IMC ≥ 30 kg/m^2^). O índice de massa corporal (IMC) foi calculado a partir do relato de medidas de peso e altura.

c)*Estilo de vida:*

- Tabagismo: não fumante, ex-fumante e fumante;
- Consumo abusivo de bebida alcoólica: sim, não. Considerou-se consumo abusivo quando consumo de cinco ou mais doses em uma única ocasião.
- Consumir cinco ou mais grupos de alimentos não ou minimamente processados protetores contra doenças crônicas no dia anterior à entrevista como: (arroz; mandioca/aipim/macaxeira; feijão, ervilha; carne de boi, porco, frango ou peixe; ovo; alface, couve, brócolis; abóbora, cenoura; tomate e legumes; mamão, manga; laranja, e frutas; leite; amendoim, castanhas);
- Consumo de alimentos ultraprocessados: sim ou não. Considerou-se o relato de consumo de cinco ou mais grupos de alimentos industrializados no dia anterior a entrevista: (refrigerante; suco de fruta em caixinha; bebida achocolatada; biscoito/bolacha salgado; biscoito/bolacha doce ou recheado; sobremesa industrializada; mortadela ou presunto; pão de forma; molhos industrializados; macarrão instantâneo);
- Ingestão elevada de sal: sim, não. Considerou-se elevado quando os indivíduos responderam “Muito alto” ou “Alto” à pergunta: “Considerando a comida preparada na hora e os alimentos industrializados, o(a) Sr(a) acha que o seu consumo de sal é...”
- Atividade física suficiente no lazer (AFL): sim; não. Considerou-se como ativos no lazer os indivíduos que relataram praticar pelo menos 150 minutos semanais de intensidade leve ou moderada ou 75 minutos semanais de intensidade vigorosa.

### Análise Estatística

Primeiramente, calculou-se a prevalência de hipertensão arterial e intervalos de 95% de confiança (IC95%), de acordo com as características sociodemográficas e de saúde. Para verificar os fatores associados a essa prevalência, usou-se como medida de associação a razão de prevalência (RP) bruta e ajustada por sexo, idade, raça/cor e escolaridade, obtida por meio da Regressão de Poisson com variância robusta.

Todas as análises foram realizadas no software Data *Analysis and Statistical Software (Stata)* versão 14, empregando-se o módulo *survey* que considera os pesos de pós-estratificação.

### Aspectos Éticos

O projeto da PNS foi encaminhado à Comissão Nacional de Ética em Pesquisa do Conselho Nacional de Saúde e aprovado sob o Parecer nº 3.529.376, emitido em 23 de agosto de 2019. O presente estudo utilizou dados secundários da PNS, sem identificação dos sujeitos e publicamente disponíveis, sendo dispensado de apreciação em comitê de ética em pesquisa, em conformidade com a Resolução CNS nº 466/2012.

## RESULTADOS

A prevalência de hipertensão arterial autorreferida, segundo diagnóstico médico prévio, foi de 23,9% (IC95% 23,5–24,4), e mostrou-se mais alta entre as mulheres (26,4%; IC95% 25,8–27,2), entre pessoas idosas, com 60 anos ou mais (55,0%; IC95% 53,9–56,1), entre a população com baixa escolaridade (36,6%; IC95% 35,7–37,5) e de raça/cor preta (25,8%; IC95% 24,4–27,2) e entre pessoas características diversas 27,7% (IC95% 23,3–32,2). As prevalências foram mais altas no grupo com renda entre um e três salários-mínimos (Tabela 1).

**Tabela 1:** Prevalência e intervalo de confiança (95%) para hipertensão arterial segundo características sociodemográficas. Pesquisa Nacional de Saúde, Brasil, 2019.

Características sociodemográficas	Hipertensão autorreferida								

	%	(IC95%)		RP	(IC95%)		RPaj	(IC95%)	
Total	23,93	23,42	24,43						
Masculino	21,06	20,37	21,75	1,00			1,00		
Feminino	26,45	25,75	27,15	1,26	1,21	1,31	**1,18**	**1,14**	**1,22**
Faixa etária (anos)									
18–24	2,29	1,73	2,85	1,00			1,00		
25–39	7,25	6,69	7,81	3,16	2,44	4,10	**3,21**	**2,47**	**4,16**
40–59	27,21	26,28	28,14	11,88	9,27	15,22	**11,52**	**8,98**	**14,77**
≥ 60	54,99	53,92	56,06	24,00	18,77	30,70	**22,17**	**17,31**	**28,39**
Escolaridade									
Sem instrução e fundamental incompleto	36,55	35,65	37,45	1,00			1,00		
Fundamental completo e médio incompleto	20,38	19,13	21,63	0,56	0,52	0,60	0,96	0,91	1,02
Médio completo e superior incompleto	15,44	14,71	16,17	0,42	0,40	0,45	**0,84**	**0,80**	**0,88**
Superior completo	18,17	17,07	19,27	0,50	0,47	0,53	**0,74**	**0,70**	**0,79**
Raça/cor									
Branco	24,36	23,56	25,16	1,00			1,00		
Pardo	22,88	22,18	23,58	0,94	0,90	0,98	**1,05**	**1,01**	**1,09**
Preto	25,81	24,42	27,19	1,06	1,00	1,13	**1,15**	**1,08**	**1,22**
Outros (amarela/indígena)	27,74	23,25	32,22	1,14	0,97	1,34	**1,15**	**1,02**	**1,30**
Plano de saúde									
Não	23,77	23,20	24,34	1,00			1,00		
Sim	24,34	23,35	25,34	1,02	0,98	1,07	**1,07**	**1,02**	**1,12**
Rendimentos (sm)									
< 1	22,42	21,76	23,07	1,00			1,00		
≥ 1–3	25,64	24,80	26,48	1,14	1,10	1,19	1,00	0,97	1,04
> 3	25,15	23,63	26,66	1,12	1,05	1,20	1,02	0,95	1,09

RP: razão de prevalência; RPaj: razão de prevalência ajustada por sexo, idade e escolaridade; SM: salário-mínimo.

Valores com significância estatística estão apresentados em negrito.

As razões de prevalência (RP) ajustadas por sexo, escolaridade e idade foram: sexo feminino (RPaj = 1,2; IC95% 1,1–1,2); faixa etária – referência: 18 a 24, 25 a 39 anos (RPaj = 3,2; IC95% 2,5–4,2), 40 a 59 anos (RPaj = 11,5; IC95% 9,0–14,8), 60 e mais (RPaj = 22,2; IC95% 17,3–28,4); escolaridade –referência: sem instrução e fundamental incompleto, com menores prevalências de hipertensão nas faixas mais altas de escolaridade, sendo para população com ensino médio completo e superior incompleto (RPaj = 0,8; IC95% 0,8–0,9), superior completo (RPaj = 0,7; IC95% 0,7–0,8); assumindo a raça/cor branca como referência, a maior prevalência de hipertensão foi entre pretos (RPaj = 1,1; IC95% 1,1–1,2), pardos (RPaj = 1,0; IC95% 1,0–1,1), outros (RPaj = 1,2; IC95% 1,0–1,3); quem tem plano de saúde (RPaj = 1,1; IC95% 1,0–1,1). Não houve diferença quanto de prevalência quanto a renda (Tabela 1).

A Tabela 2 apresenta as prevalências de hipertensão, segundo as condições clínicas. A prevalência de hipertensão foi mais alta entre: quem avalia a sua situação de saúde como regular e ruim a 38,2% (IC95% 37,2–39,3), e 49,8% (IC95% 47,6–52,0), respectivamente; quem tem doença do coração 61,2% (IC95% 59,0–64,0), diabetes 63,8% (IC95% 61,9–65,7), colesterol elevado 50,9% (IC95% 49,4–52,3), sobrepeso 25,7% (IC95% 24,9–26,5) e obesidade 37,2% (IC95% 36,0–38,4).

**Tabela 2:** Prevalência e intervalo de confiança (95%) para hipertensão arterial segundo condições clínicas. Pesquisa Nacional de Saúde, Brasil, 2019.

Estilo de vida	Hipertensão autorreferida								

	%	(IC95%)		RP	(IC95%)		RPaj	(IC95%)	
Tabagismo									
Não fumante	20,59	19,98	21,20	1,00			1,00		
Ex-fumante	32,91	31,85	33,96	1,60	1,53	1,67	**1,12**	**1,08**	**1,16**
Fumante	21,04	19,66	22,43	1,02	0,95	1,09	**0,88**	**0,82**	**0,94**
Cinco ou mais grupos de alimentos protetores									
Não	23,30	22,73	23,88	1,00			1,00		
Sim	25,93	24,92	26,93	1,11	1,06	1,16	0,98	0,94	1,02
Cinco ou mais grupos de alimentos ultraprocessados									
Não	25,53	24,97	26,10	1,00			1,00		
Sim	14,30	13,23	15,37	0,56	0,52	0,61	**0,86**	**0,80**	**0,93**
Consumo abusivo de bebidas alcoólicas									
Não	25,56	24,99	26,13	1,00			1,00		
Sim	15,98	14,89	17,06	0,63	0,58	0,67	1,03	0,97	1,10
Ingestão elevada de sal									
Não	24,61	24,07	25,15	1,00			1,00		
Sim	19,23	17,91	20,55	0,78	0,73	0,84	**1,08**	**1,01**	**1,14**
Prática de atividade física no tempo livre									
Não	26,22	25,61	26,83	1,00			1,00		
Sim	18,44	17,56	19,32	0,70	0,67	0,74	0,98	0,93	1,03

RP: razão de prevalência; RPaj: razão de prevalência ajustada por sexo, idade e escolaridade.

Valores com significância estatística estão apresentados em negrito.

Ao ajustar por idade, sexo e escolaridade as RPaj, foram mais altas entre: quem avalia a situação de saúde como regular (RPaj = 1,6; IC95% 1,5–1,6) e ruim (RPaj = 1,7; IC95% 1,6–1,8); quem diz ter doença do coração (RPaj = 1,7; IC95% 1,6–1,7), diabetes (RPaj = 1,7; IC95% 1,6–1,8), colesterol alto (RPaj = 1,6; IC95% 1,6–1,7); sobrepeso (RPaj = 1,4; IC95% 1,4–1,5); e obesidade (RPaj = 2,0; IC95% 1,9–2,1).

A Tabela 3 apresenta as prevalências de hipertensão arterial conforme estilos de vida: entre ex-fumantes 32,9% (IC95% 31,9–34,0), fumantes 21,0% (IC95% 19,7–22,4); quem consome alimentos saudáveis 25,9% (IC95% 24,9–26,9), e foi mais baixa entre quem consome alimentos ultraprocessados: 14,3% (IC95% 13,2–15,4); entre quem faz uso abusivo de bebidas alcoólicas 16,0% (IC95% 15,0–17,0); consumo elevado de sal 19,2% (IC95% 18,0–20,6), e prática de atividade física 18,4% (IC95% 17,6–19,3).

**Tabela 3:** Prevalência e intervalo de confiança (95%) para hipertensão arterial segundo condições clínicas. Pesquisa Nacional de Saúde, Brasil, 2019.

Condições clínicas	Hipertensão autorreferida								

	%	(IC95%)		RP	(IC95%)		RPaj	(IC95%)	
Avaliação do estado de saúde									
Bom/muito bom	16,06	15,54	16,59	1,00			1,00		
Regular	38,23	37,22	39,25	2,38	2,28	2,48	**1,56**	**1,50**	**1,62**
Ruim/muito ruim	49,77	47,57	51,97	3,10	2,94	3,27	**1,74**	**1,65**	**1,83**
Doença cardíaca									
Não	21,84	21,33	22,35	1,00			1,00		
Sim	61,23	58,96	63,50	2,80	2,68	2,93	**1,67**	**1,60**	**1,74**
Diabetes									
Não	20,59	20,10	21,08	1,00			1,00		
Sim	63,75	61,85	65,66	3,10	2,98	3,21	**1,69**	**1,63**	**1,76**
Colesterol elevado^a^									
Não	20,50	19,97	21,03	1,00			1,00		
Sim	50,85	49,39	52,32	2,48	2,39	2,57	**1,63**	**1,57**	**1,69**
Estado nutricional^b^									
Eutrófico	16,07	15,40	16,74	1,00			1,00		
Sobrepeso	25,69	24,86	26,51	1,60	1,52	1,69	**1,42**	**1,36**	**1,49**
Obesidade	37,20	36,01	38,39	2,32	2,20	2,44	**2,02**	**1,92**	**2,12**

RP: razão de prevalência; RPaj: razão de prevalência ajustada por sexo, idade e escolaridade.

^a^ 7.280 *missing.*

^b^ 853 *missing*.

Valores com significância estatística estão apresentados em negrito.

Ao ajustar por idade, sexo e escolaridade as RPaj, foram mais altas entre: consumo elevado de sal (RPaj = 1,1; IC95% 1,0–1,1); ex-fumantes (RPaj = 1,1; IC95% 1,1–1,2); e menor entre fumantes (RPaj = 0,9; IC95% 0,8–0,9); quem consome alimentos ultraprocessados (RPaj = 0,9; IC95% 0,8–0,9). As associações entre consumo de alimentos saudáveis, consumo abusivo de bebidas alcoólicas e prática suficiente de atividade física com a hipertensão não foram significativas no modelo ajustado.

## DISCUSSÃO

A hipertensão arterial autorreferida foi declarada por cerca de um quarto da população, sendo mais alta entre as mulheres, com faixas etárias mais elevadas, mais alta também na população com baixa escolaridade, entre pretos e pardos, e associa-se ainda a comorbidades como: doença do coração, diabetes, colesterol elevado, sobrepeso, obesidade, e pessoas que avaliam a saúde como regular ou ruim. Entre os estilos de vida, a prevalência mais alta foi entre ex-fumantes, que referem consumo elevado de sal, e foi mais baixa entre fumantes, e quem faz uso abusivo de bebida alcoólica.

As prevalências autorreferidas da PNS 2013 foram mais baixas, 21,4% (IC95% 20.8–22.0)^9^, do que foi encontrado no presente estudo, o que pode ser explicado pelo envelhecimento populacional, além do aumento da prevalência de obesidade e excesso de peso na população no período.

Quanto aos fatores sociodemográficos, observaram-se prevalências mais altas de hipertensão arterial entre as mulheres. Segundo estudo^9^, as mulheres tender a procurar mais os serviços de saúde que os homens, o que lhes dá maiores oportunidades de um diagnóstico, esse fato pode ter se refletido na prevalência de hipertensão arterial mais acentuada entre os entrevistados do sexo feminino. Entretanto, ao utilizar medidas aferidas de pressão arterial, a PNS 2013 identificou o inverso: pressão mais elevada entre os homens^10^, o que também foi observado em uma metanálise realizada a partir de 1.479 estudos – reunidos, representam a medida de pressão arterial de 19,1 milhões de adultos, número estimado pelo grupo de estudos globais em 2015 *(NCD Risk Factor Collaboration)* –, que estimou prevalência de hipertensão em 24,1% dos homens (IC95% 21,4–27,1) e em 20,1% das mulheres (IC95% 17,8–22,5)^8^.

A literatura destaca que a hipertensão arterial aumenta com a idade, pois se associa ao envelhecimento e ao seu progressivo enrijecimento dos vasos sanguíneos^1,3,11^. Destaca-se que a idade foi o fator de maior magnitude de associação neste trabalho.

O estudo identificou, após ajustes por idade, sexo e escolaridade, prevalências mais elevadas entre pretos, pardos, amarelos e indígenas. A literatura destaca que a hipertensão arterial pode ser resultante de uma predisposição genética em negros, além de ser uma população mais exposta em termos sociais aos fatores de risco inerentes à hipertensão^12^.

Quanto às características socioeconômicas, populações com baixa escolaridade apresentaram maior incidência da hipertensão arterial. Dentre os motivos para esse resultado, diversos estudos apontam para a exposição a vulnerabilidades e fatores estressores, piores condições socioeconômicas, falta de acesso aos serviços de saúde, menor acesso a orientações, ações de promoção a saúde, menor oportunidade para acesso a alimentação saudável, e cuidados em saúde^13^. Entretanto, após o controle das variáveis de ajuste, a renda não mostrou associação à condição clínica focalizada neste estudo. Pessoas que contrataram planos de saúde apresentaram resultado inverso, o que já fora indicado em outros estudos no Brasil^14^. Ter um plano de saúde contratado acaba funcionando como um facilitador de acesso a serviços de saúde, o que proporciona maior oportunidade de diagnóstico.

Fatores que incidem sobre o estilo de vida da população, como o uso de tabaco, são considerados fontes de risco para doenças cardiovasculares^15^ e, para evitar esses agravos, a cessação do consumo dessas substâncias é a primeira recomendação para sua prevenção^3^. A associação entre cessação do hábito de fumar e hipertensão arterial já foi descrita na literatura^11^ e pode ser tanto efeito do delineamento transversal do estudo quanto pelo abandono do vício após orientação médica a partir do diagnóstico3. Porém, ao lado do ganho de peso, o abandono do tabagismo é descrito como um possível fator estressante, o que pode, ao contrário, contribuir para aumentar o risco de hipertensão.

Outro importante fator de risco para a prevalência de hipertensão arterial é o consumo abusivo de sal, o que se confirmou neste estudo, mesmo após os ajustes. A recomendação geral para consumo de sal é de 2g ou 5g de sódio por dia^16^. Estudo de metanálise realizado na China mostrou que a substituição do sal de mesa por cloreto de potássio pode trazer benefícios na redução da pressão arterial sistólica (-5,7mmHg; IC95% -8,5 a -2,8) e a pressão arterial diastólica (-2,0mmHg; IC95% -3,5 a -0,4)^17^.

Estudos longitudinais e revisões sistemáticas^18^ apontam que uma alimentação rica em frutas, vegetais, grãos e baixo teor de gordura podem resultar em redução na pressão arterial e a adesão à dieta com maior teor de frutas, verduras e hortaliças está associada a menor risco de acidente vascular encefálico^18^, mortalidade cardiovascular^19^ e doença renal crônica^20^. Da mesma forma, todas as evidências da literatura indicam que alimentos com elevado teor de gordura, sal, aditivos, aumentam as prevalências de doenças cardiovasculares e de doenças crônicas não transmissíveis^16^. Entretanto encontramos neste estudo que pessoas com dieta não saudável teriam menor prevalência de hipertensão arterial, um resultado não esperado. Porém as análises complementares, não mostradas, identificaram que o consumo de alimentos ultraprocessados foi até quatro vezes mais elevado em populações mais jovens, com idades entre 18 e 34 anos, comparada com idosos, que por sua vez tem prevalências de hipertensão até 20 vezes mais elevadas. Portanto, mesmo ajustando por idade e outras variáveis do modelo, o efeito persistiu, possivelmente pela força da associação desse tipo de alimento entre jovens e os limites dos estudos transversais, nas análises de causalidade^21^.

Da mesma forma, as evidências de que a prática regular de atividades físicas (150 min/semana) e a redução do comportamento sedentário reduzem a hipertensão arterial são claras na literatura^22^. Estima-se que o consumo excessivo de álcool seja responsável por cerca de 10% a 30% dos casos de hipertensão e por aproximadamente 6% da mortalidade de todas as causas no mundo^23,24^. Neste estudo, entretanto, as associações com atividade física, alimentação saudável e álcool, não foram identificadas, o que também já foi descrito em outros estudos transversais, analisando resultados de inquéritos por telefone e hipertensão^7^. O fato de que, populações jovens têm maior frequência de atividade física, álcool e também de alimentos ultraprocessados, pode justificar esse resultado negativo, mesmo após ajuste por idade.

A hipertensão é o principal fator de risco modificável com associação para doenças cardiovasculares, doenças renais crônicas e morte prematura. Associa-se a fatores de riscos metabólicos para as doenças dos sistemas cardiocirculatório e renal, como dislipidemia, obesidade abdominal e DM^3^.

Este estudo apontou ainda que as comorbidades, como a obesidade, foram a variável com maior força de associação com a hipertensão arterial A literatura aponta uma relação direta, contínua e quase linear entre o excesso de peso (sobrepeso/obesidade) e os níveis de pressão alta^25^. A obesidade, em especial a visceral, constitui um importante fator de risco para a elevação da hipertensão, podendo ser responsável por 65% a 75% dos casos de pressão alta^26^. Uma das medidas mais importantes no controle da doença é a redução de peso, como identificou uma metanálise incluindo 25 estudos, apontando que a perda de 5,1 kg reduziu, em média, a pressão arterial sistólica em 4,4 mmHg e a diastólica em 3,6 mmHg^25^.

Estudos têm demonstrado que a elevação da pressão arterial resulta no aumento de riscos de insuficiência cardíaca, fibrilação atrial, cardiopatias valvares e doença arterial periférica^24^, doença renal crônica^,24^, demência e doença de Alzheimer^27^ e, provavelmente, diabetes mellitus^28^. Esses desfechos ocorrem em decorrência de muitos anos de exposição aos níveis pressóricos elevados^24^. O estudo atual, embora de delineamento transversal, confirmou a associação com diabetes e doença cardiovascular, colesterol elevado e obesidade.

A autoavaliação de saúde ruim ou muito ruim mostrou-se fortemente associada com a hipertensão arterial. Estudos apontam esse indicador como um preditor de piores desfechos em saúde e mortalidade^29^.

Dentre as limitações deste estudo, por se tratar de desenho de estudo transversal, estão as aferições dos fatores de risco ou de proteção e dos desfechos que são realizadas simultaneamente, o que limita inferências sobre a direcionalidade de algumas associações no modelo causal. Destaca-se o viés da causalidade reversa ou mudanças dos estilos de vida, motivas pelo diagnóstico da doença e pelas orientações dos profissionais de saúde. A utilização de dados de morbidade autorreferida é dependente do acesso aos serviços de saúde para o diagnóstico; assim, usuários que utilizam mais o serviço têm maior oportunidade do diagnóstico médico de hipertensão. Por outro lado, por se tratar da mais importante pesquisa se saúde no país, os dados permitem o monitoramento desse agravo na população brasileira.

Em síntese, os resultados estimam que cerca de um quarto da população adulta brasileira afirmou ter hipertensão arterial, desfecho mais prevalente em mulheres e mostrando-se associada às faixas etárias mais elevadas, com a raça/cor da pele preta e parda, baixa escolaridade, consumo elevado de sal, ex-tabagismo, presença de comorbidades e pior autoavaliação de saúde. Conhecer esses fatores pode ajudar na prevenção, identificação, manejo e controle da hipertensão arterial e das doenças crônicas não transmissíveis.
